# Child-Centered Care: A Qualitative Study Exploring Pediatric Hospitalization Through Children’s Perspectives

**DOI:** 10.3390/nursrep14040228

**Published:** 2024-10-22

**Authors:** Amalia Sillero Sillero, Raquel Ayuso Margañon, Elena Marques-Sule, María Gil Poisa

**Affiliations:** 1 Escoles Universitàries Gimbernat, Adscrites a Universitat Autònoma de Barcelona, 08174 Sant Cugat, Spain; 2 Social Determinants and Health Education Research Group (SDHEd), Hospital del Mar Research Institute, 08003 Barcelona, Spain; mgilp@esimar.edu.es; 3 Hospital del Mar Nursing School (ESIHMar), Universitat Pompeu Fabra-Affiliated, 08003 Barcelona, Spain; 4 Physiotherapy in Motion, Multispeciality Research Group (PTin MOTION), Department of Physiotherapy, University of Valencia, 46010 Valencia, Spain; elena.marques@uv.es

**Keywords:** pediatric hospitalization, child anxiety, family-centered care, thematic analysis, patient experience, healthcare communication, emotional support

## Abstract

**Background/Objectives**: Hospitalization can be stressful for children due to the influence of unfamiliar environments, separation from family, and interactions with healthcare professionals. This study aimed to explore children’s hospitalization experiences from a child-centered care perspective to develop interventions that better meet their emotional and psychological needs. **Methods**: This qualitative study employed Husserl’s descriptive phenomenology to explore hospitalization experiences among children aged 9–13 years. Conducted at a primary health center in Spain from October 2022 to June 2023, the study used purposive sampling to select participants hospitalized within the past six months. Data were collected through in-depth interviews and children’s self-created drawings, analyzed using thematic analysis with ATLAS.ti software. **Results**: A total of 10 school-age children (five boys and five girls) were interviewed. Three main themes emerged: (1) Emotions and Feelings—children described fear, anxiety, and loneliness during their hospital stay; (2) Experiences of Pain and Discomfort—participants reported physical pain and discomfort; (3) Interactions with Medical Staff—children expressed a need for more detailed explanations and clearer communication. **Conclusions**: The findings highlight the importance of family-centered care and improved communication between healthcare providers and children. Strategies like art therapy and flexible visiting hours could provide better emotional support. Tailored communication to children’s developmental levels is crucial. Integrating these strategies into clinical practice could enhance the well-being of hospitalized children. Future research should focus on evaluating these interventions to improve pediatric care.

## 1. Introduction

The experience of hospitalization can be profoundly challenging for children, often marked by stressors such as an unfamiliar environment, separation from family and friends, and interactions with healthcare professionals [1,2]. These elements frequently contribute to heightened anxiety and stress, which are further intensified by physical pain, immobility, and a perceived loss of control [3,4]. A child’s prior experiences with illness, the severity of their current condition, and their emotional responses are critical factors that shape their overall hospitalization experience [5].

Understanding these experiences necessitates examining the role of expectations in shaping children’s hospital encounters. Expectation–Confirmation Theory [6] posits that a child’s and their parent’s pre-existing expectations—such as anticipated pain, treatment quality, and healthcare professional behaviour—are pivotal in determining their overall satisfaction. When hospital experiences align with or surpass these expectations, children are more likely to have a positive experience. However, a disparity between expectations and reality can result in increased fear, anxiety, and dissatisfaction. These expectations are not formed in a vacuum but are influenced by various environmental factors, as outlined in Bronfenbrenner’s Ecological Systems Theory [7]. A child’s family, previous healthcare experiences, media portrayals, and broader cultural contexts shape their expectations. For instance, negative media representations of hospitals or distressing stories from family members may lead to heightened anxiety before hospitalization [8].

Moreover, Cognitive Development Piaget Theory [9] highlights how a child’s stage of cognitive development influences their understanding of hospitalization. Younger children may engage in magical thinking, leading to misconceptions about their medical situation. In comparison, older children, though more capable of understanding, might still struggle with anxiety related to perceived loss of control or fear of pain [4].

Child-centered care, a fundamental principle in paediatric care, emphasizes the importance of considering children’s individual perspectives and needs in medical care. This theoretical approach stresses that children are not simply passive recipients of care, but active participants whose opinions and experiences should be valued and respected. Child-centered care involves tailoring healthcare practices to meet the unique needs of each child, ensuring their voices are heard and their rights are upheld. This model advocates for the inclusion of children in decision-making processes, respecting their views and providing age-appropriate information to help them understand their medical conditions and treatments [8]. Understanding children’s experiences of illness and hospitalization—considering the influence of their cognitive development, environmental context, and expectations—is crucial for effective healthcare management [3]. Children’s perceptions of their hospital experiences provide valuable information for improving their adjustment to chronic illness and refining hospital services [10]. By listening to children’s thoughts and feelings and encouraging open expression, healthcare providers can better support their ‘right to participate’ in healthcare decisions, improving the quality of care and optimizing health services [11]. Implementing child-centered care not only enhances the hospital experience for children but also promotes their overall well-being and recovery [8].

Despite the importance of understanding children’s perspectives, much of the existing research has primarily focused on children with chronic illnesses, often gathering data from parents and healthcare providers [12,13]. There is a relative paucity of studies that directly address children’s own experiences, where they are considered active participants rather than passive users of the healthcare system [3,10,14]. Children are often silent in research, with their perceptions frequently viewed merely as a developmental stage. Some authors argue that this silence stems from a societal desire to protect their well-being [5]. Filling this gap is crucial to developing interventions that truly respond to the emotional and psychological needs of hospitalized children, thus improving their experience and health outcomes.

This study, therefore, seeks to focus the child’s perspective, engaging them directly to articulate their experiences, wishes, and desires regarding their hospitalization. By prioritising children’s views, this research aligns with the principles of the United Nations Convention on the Rights of the Child [15], emphasizing their right to participate in decisions affecting their care. This approach is vital for moving beyond traditional outcomes and towards more child-centered care, ultimately enhancing the quality of pediatric healthcare services. By adopting child-centered care, this research underscores the importance of viewing children as active participants in their healthcare, ensuring their voices are heard and respected in the development of care practices.

Therefore, this study aims to explore children’s hospitalization experiences from a child-centered care approach, to generate preliminary insights that guide future research and clinical practice in developing interventions that better meet the emotional, psychological, and overall needs of hospitalized children.

## 2. Materials and Methods

### 2.1. Study Design and Sample

This qualitative study utilizes Husserl’s descriptive phenomenology [16] to explore children’s experiences of hospitalization related to their illness care. Data were collected through in-depth interviews using a question guide, as well as from the children’s explanations and drawings.

This study was conducted at a primary health centre in Spain from October 2022 to June 2023. A purposive sampling method was employed, focusing on children aged 9–13 who had been hospitalized at the designated medical centre within the past six months. The selection of this age range was based on the children’s ability to communicate clearly and reflect on their experiences. In addition, this helped to maintain the homogeneity of the group, which facilitated the analysis of the data. Eligibility requires caregiver consent, child assent, adequate cognitive and communication abilities, and stable health status. Exclusion criteria were children who had been hospitalized for more than 30 consecutive days in the last six months, as their experience might have significantly differed, those who were participating in other research studies on hospitalization to avoid data biases, children with severe psychological disorders that could have impaired their ability to effectively participate in interviews, and those who had critical or unstable health conditions. Children were contacted by the primary care centre nurse during routine follow-up visits after hospital discharge. The nurse identified potential participants, provided information about the study to the caregivers and children, and obtained their consent and assent, respectively. This approach ensured that participants were selected based on specific criteria relevant to the study’s objectives. Permission was obtained from the child and their caregivers to participate in the study. The sample size was not pre-determined; instead, the first available cases meeting these criteria were selected to ensure a rich dataset. Data collection continued until information saturation was achieved, meaning no new themes or insights were emerging from the interviews. This approach allowed the sample size to be adjusted according to the quality and depth of the data obtained, rather than a fixed number of participants. This approach ensured that the number of participants was sufficient to provide comprehensive and in-depth data for the study. The final sample included ten children, evenly distributed between five boys and five girls, aged 9 to 13 years who had been hospitalized for at least three days and were no longer hospitalized during the interview.

### 2.2. Data Collection

Data collection occurred in the children’s homes, offering the participants a comfortable and familiar environment. It was carried out by nurses with extensive experience in both clinical and qualitative research. This facilitated the establishment of a trusting relationship with the participants and ensured a better understanding of the participants’ responses, as well as a greater ability to ask relevant questions. Before the researcher’s visit, each child was asked to create a drawing depicting their hospital experience. This creative and engaging method provided a more profound insight into the children’s experiences. During the home visit, the child was asked to explain the meaning of their drawing in their own words, and their explanations were recorded for later transcription. The drawing technique facilitated rapport between the researcher and the child and provided a natural introduction to the interview. The drawings often led to biographical questions, such as: “What is happening here?”, “What does this mean to you?”, and “How does this make you feel?”. Additionally, specific questions were asked about these aspects during the interview to understand their emotions, feelings about their social and physical environment, what they wished they could do, how they communicated and interacted, and the physical care they received. These questions helped the children express their feelings and experiences more openly. The interview questions were designed based on a thorough review of the literature on pediatric patient experiences and qualitative research methods. Studies, such as those by Demirbağ et al. [11], de Man et al. [12], and de Manet et al. [13] guided our approach. We aimed to ask open-ended, child-friendly questions to capture children’s emotions and perceptions effectively []. Additionally, we considered the child-centered care principle, encouraging children to directly share their experiences and wishes regarding their hospitalization [10]. See Table 1. The drawings created by the children served as a tool to guide the interviews, helping the researchers establish a connection with the participants and delve deeper into their hospital experiences. However, the themes emerged directly from the transcribed interviews and not from the drawings themselves. This approach allowed for a richer and more nuanced interpretation of the children’s experiences.

Interviews were meticulously managed to last between 20 and 30 min, ensuring the children were not fatigued. This approach, which prioritized the children’s comfort and well-being, reassured the participants and their caregivers about the ethical conduct of the study, enabling them to engage in the interview fully. Parents were present during the interviews to provide support and consent. However, their presence was considered as a potential source of response bias, and efforts were made to minimize this by encouraging children to express their own views independently.

### 2.3. Data Analysis

The research employed thematic analysis, utilizing an inductive approach for data analysis. In thematic analysis, themes are not predetermined but emerge organically from the data, allowing for a more nuanced interpretation [17].

The data collection process involved recording the interviews and transcribing them verbatim. Initially, author A.S.S. independently transcribed the audio recordings within 24 h of each interview’s completion. Following this, authors R.A.M. and M.P. independently reviewed the transcripts multiple times to ensure thorough understanding and accuracy, instilling confidence in the research findings. Thematic analysis was performed using ATLAS.ti software, version 22 (ATLAS.ti Scientific Software Development GmbH, Berlin, Germany). The process began with generating initial codes, refined into themes and categories through systematic analysis. This process was guided by a comprehensive literature review, which informed the development of emerging themes and categories.

The authors collaboratively discussed and revised the final themes, ensuring they accurately reflected the participants’ experiences. The analysis report was then compiled, selecting extracts that most effectively represented the participants’ perspectives in alignment with the research question and existing literature.

### 2.4. Trustworthiness

This process adheres to credibility, transferability, dependability, and confirmability criteria in qualitative data collection and analysis, following literature guidelines [18]. To ensure comprehensive and transparent reporting, we adhered to the Standards for Reporting Qualitative Research (SRQR) guidelines throughout this study [19]. The SRQR checklist is provided as supplementary material (see Supplementary Material Table S1). Once thematic redundancy was achieved, an additional child was recruited to confirm data saturation. A doctoral-prepared certified child paediatrics nurse practitioner was consulted as a peer debriefer to review transcripts and themes. Investigator and theoretical triangulation criteria were established based on referenced contributions [20].

### 2.5. Theoretical Framework and Research Team Statements

Using Husserl’s descriptive phenomenology approach [16], we aimed to explore lived experiences, ensuring the discovery of new knowledge while avoiding undue influence on participants’ understanding of the phenomenon. Adhering to the four bracketing strategies proposed by Chan et al. [21]—mental preparation, scope definition in the literature review, planning data collection, and planning data analysis—we prioritized rigor in our research. This study spent significant time before and during interviews to develop relationships and maintain prolonged engagement with each child.

### 2.6. Ethical Considerations

The research adhered to international ethical standards, including the World Medical Association Declaration of Helsinki [22] and Spanish Law 14/2007 on Biomedical Research, classifying it as non-experimental due to the absence of biomedical interventions. The Ethics Committee of Research in Humans of the Ethics Commission in Experimental Research approved this Study (Approval Code: 1544058). Informed consent was obtained from minors and their guardians, with an impartial witness present when necessary. Contact with recently hospitalized children was facilitated through nurses during their routine check-ups. Ethical measures ensured consent from children and their caregivers, confidentiality with fictitious names, and secure data handling in compliance with GDPR (Regulation (EU) 2016/679) and Organic Law 3/2018 on data protection. Interviews were recorded, transcribed, reviewed by participants, and subsequently deleted.

## 3. Results

### 3.1. Participants Profile

A total of 10 school-age children (five boys and five girls) were interviewed about their hospitalization experiences. The average age of the participants was 11.7 years (SD = 1.55), and the average length of hospitalization was eight days (SD = 2.83); the diagnoses were asthma, abdominal pain, pneumonia, bronchitis, migraine, peritonitis, traumatic brain injury, and appendicitis. See Table 2.

### 3.2. Thematic Findings

The children’s drawings, used to initiate the interviews, often aligned with their verbal explanations, vividly illustrating their experiences. These drawings served as a medium for expressing their emotions and experiences. The insights gained from the drawings and interview responses were categorized into three mains themes and nine subthemes. These psychological and emotional needs are intrinsically linked to other aspects of hospitalization, such as physical care, entertainment and interaction, communication/information, and the social and physical environment. To provide a comprehensive view of these experiences, we have adopted a holistic approach that considers all these dimensions. See Table 3.

### 3.3. Emotions and Feelings

This theme includes four subthemes that describe the children’s emotional experiences during their hospitalization, highlighting their fears, anxieties, and feelings of loneliness.

#### 3.3.1. Fear of Death and Unfamiliar Hospital Setting

Some children expressed a profound fear about the potential outcomes of their hospital stay, including the fear of death and the unknown aspects of their treatment. This fear was heightened by their lack of understanding about their condition and what was happening to them. The unfamiliar hospital setting and the prospect of being away from home contributed to anxiety about the experience, including concerns about potential pain and prolonged separation from loved ones. The hospital environment, coupled with physical pain and fatigue, led to feelings of sadness.

“When I thought about it for a while, I was afraid that I might die”.

(Participant 1)

“When I went into the hospital, I didn’t know what was going to happen, if I would be away from home for many hours, and especially if it would hurt”.

(Participant 2)

“Being hospitalized is still sad because you’re in pain, without friends, and even though your mom is there, you’re so tired that she’s sad too. But… well, some nurses try to help us; some smile at me and squeeze my hand. There was one who sometimes read me a story”.

(Participant 6)

#### 3.3.2. Anxiety About Medical Equipment

The sight and sensation of medical equipment, such as wires and tubes, caused significant anxiety. These objects often frightened the children because they did not understand their purpose.

“I was very nervous there were so many wires, and they frightened me because I didn’t know what they were for”.

(Participant 10)

#### 3.3.3. Sensory Overload

Some children experienced sensory overload, leading to hallucinations or confusion, exacerbated by the hospital environment, including noise and unfamiliar stimuli.

“Sometimes I saw many colours, clowns, strange shapes, and sometimes I saw nothing. Nobody helped me; someone scolded me for not moving because the wires were pulling, and I didn’t want to do anything”.

(Participant 4)

#### 3.3.4. Loneliness and Isolation:

Despite the presence of medical staff, children often felt isolated and lonely, missing their family members and feeling cut off from their usual social interactions.

“I was alone in the bed with many people around, but I felt lonely. I saw how the doctors and nurses spoke kindly to my mom, but the worst part was that my siblings couldn’t come in to see me”.

(Participant 6)

### 3.4. Experiences of Pain and Discomfort

This theme highlights the physical pain and discomfort that the children experienced, which was often compounded by their inability to communicate effectively.

#### 3.4.1. Disturbances from Hospital Environment

The hospital environment posed challenges for the children, including difficulty sleeping due to constant noises and a lack of engaging activities, which contributed to their overall discomfort.

“Sometimes I was falling asleep or something, and then it would start beeping a lot; there was so much noise, I didn’t like it. It was on the night I was in intensive care, which was the worst because I was in a lot of pain”.

(Participant 5)

“Well, most of the time, I slept because there wasn’t much to do, and there was no one to talk to. They were always very busy, and it was tough to sleep because of the lights and constant adjustments, which was unbearable”.

(Participant 7)

#### 3.4.2. Medical Procedures and Improper Positioning

Medical procedures, such as the insertion of tubes, caused physical discomfort and added to the children’s distress. Moreover, uncomfortable positioning in bed led to additional pain.

“The tube in my chest was very heavy”.

(Participant 7)

“Yes, it also hurt because when I wasn’t in a good position, I would start feeling horrible pain, and until it ended, I couldn’t speak or see well. But when the nurse came in and talked to me, I felt better, I don’t know why, but that’s the truth”.

(Participant 8)

“I think they were coming in all the time, but I didn’t know what they were doing. It hurt, then it didn’t, but I didn’t understand anything, I didn’t see my parents, and I didn’t know what they were doing to me”.

(Participant 2)

### 3.5. Interaction and Communication with Medical Staff

This theme addresses the impact of interactions with medical staff and the importance of clear and empathetic communication in improving the children’s experiences.

#### 3.5.1. Communication Inability

Medical equipment, such as tubes and machines, hindered the children’s ability to communicate their needs, leading to feelings of frustration and helplessness.

“I couldn’t tell anyone anything because of the tube, and sometimes I needed to talk. I always wanted to hold hands, but I couldn’t say it. And with that tube, I felt pain. To communicate, I made signs, and those machines tortured me”.

(Participant 9)

#### 3.5.2. Lack of Information

Children expressed a desire for clearer explanations about their treatment and medical procedures, which would help them understand what was happening to them.

“I think they were always coming in, but I didn’t know what they were doing. It hurt, then it didn’t, but I didn’t understand anything, I didn’t see my parents, and I didn’t know what they were doing to me”.

(Participant 2)

“One understands that there are issues, but it would be good if someone could explain how everything is going”.

(Participant 3)

#### 3.5.3. Positive Interactions with Nurses

Positive interactions with nurses, including their explanations and affectionate care, were appreciated and had a comforting effect on the children. Moreover, children valued the attentiveness and supportive behavior of the nursing staff, who provided comfort and tried to address their emotional and physical needs.

“My drawing is of a nurse because I really liked how the nurses treated me and the others. They were always attentive, if my fluid was running out, if I cried, or if I was misbehaving with my mom. They always had something to say to keep me from crying or when I was in pain”.

(Participant 5)

“I saw how the nurses, when their shift ended, explained very well to the other nurses what they needed to do and talked about me and my family, but they did it with affection, I think”.

(Participant 4)

## 4. Discussion

This study aimed to explore children’s experiences during hospitalization, from revealing significant emotional and psychological challenges associated with child-centered care to developing interventions that better meet their emotional and psychological needs. To provide a comprehensive view of these experiences, we have adopted a holistic approach that considers different dimensions related to other aspects of hospitalization, such as physical care, entertainment and interaction, communication/information, and the social and physical environment. The findings underscore that hospitalization is not only a physical challenge but also an emotionally draining experience for children, characterized by fear, anxiety, and feelings of isolation. These results are consistent with existing literature that describes similar emotional responses in pediatric populations [2,23,24].

In terms of emotional impact, children’s drawings and verbal accounts vividly illustrate their feelings of loneliness, fear, and anxiety, reflecting the profound emotional impact of hospitalization. This emotional upheaval aligns with previous research indicating that hospitalization is not merely a physical experience but also an emotionally exhausting one [25,26]. Innovative approaches such as art therapy and interactive emotional support tools could be crucial in addressing these emotional challenges. These interventions have proven effective in promoting emotional expression and improving children’s psychological well-being in hospital settings [27]. For instance, art therapy is an effective tool for reducing anxiety and providing an outlet for the complex emotions children experience [28].

Regarding the need for parental presence, a key finding of the study was children’s need to spend more time with their parents during hospitalization. This finding underscores the importance of family-centered care and aligns with research highlighting the necessity of parental presence to support children’s emotional well-being during hospitalization [8]. The literature suggests that hospital policies allowing flexible visiting hours and utilizing technology to maintain family connections could be beneficial in improving children’s experiences [5,11]. Innovations such as virtual reality and video communication have shown significant potential in maintaining family contact and providing emotional support when physical presence is not possible [29].

When it comes to communication, effective communication between healthcare professionals and children proved to be a crucial factor in alleviating fear and anxiety. Although children appreciated the efforts of the nursing staff, they expressed a desire for more detailed explanations and clear information about their care [30]. This finding highlights the need for age-appropriate communication strategies and better training programs for healthcare professionals. Interactive communication aids and regular updates on the care process could significantly improve children’s understanding and reduce their anxiety [24,31]. The literature supports the need to tailor communication to address the cognitive and emotional needs of pediatric patients, suggesting that clearer and more empathetic communication can enhance the patient experience [32].

In terms of perception, reviewed studies reveal that children perceive hospitalization as a dichotomy between the healthy world represented by family and the sick world represented by medical professionals and the hospital environment. Effective care involves bridging these two worlds through empathy, attention, and active listening [33]. The presence of patient companions is vital, as they provide essential emotional support and contribute to the child’s perception of care [1,11]. The literature also indicates that the presence of companions can moderate the emotional impact of hospitalization and improve the perceived quality of care [34].

Finally, regarding adaptation challenges, both children and their companions face challenges in adapting to the hospital environment, which can affect the overall balance and well-being of the family [35]. Research indicates that play can effectively manage feelings of loneliness and isolation, and that creative play provides emotional relief [36,37]. Furthermore, the cognitive and emotional development of children implies that they may experience complex feelings, such as guilt for their illness, which can exacerbate stress and trauma [38]. To address these concerns, it is necessary to listen carefully to children and allow them to express their feelings, focusing on the integration of adaptive and supportive interventions.

Some of these experiences have been validated in other scales, such as the Pediatric Quality of Life Inventory (PedsQL), which includes specific modules to assess satisfaction with medical care in hospitalized children and covers various domains, including physical care, entertainment, education/information, and the social and physical environment [39]. Similarly, the “Child Drawing: Hospital” scale uses playful strategies to evaluate anxiety and satisfaction in hospitalized children [40]. Our findings align with the multifaceted nature of children’s hospital experiences highlighted by these scales, reinforcing the importance of a holistic approach to address their comprehensive needs. By focusing on child-centered care, we aim to tailor interventions to meet the unique needs of each child, ensuring their voices are heard and respected in the development of care practices.

### 4.1. Limitations and Strengths

The study presents several limitations that should be acknowledged. Firstly, the results are derived from personal statements made by children and the specific environmental characteristics of the study location, which may introduce bias. Future research should include larger samples and diverse contexts to strengthen the robustness of the results. Additionally, the small sample size further limits the representativeness of the findings, as it does not encompass the broader population of hospitalized children. Lastly, the qualitative research method restricts the generalizability of the results, making them specific to the study context. Despite these limitations, the study offers several strengths contributing to its value. Qualitative research provides in-depth insights into hospitalized children’s personal experiences and perspectives, which might not be captured through quantitative methods. The study’s focus on a specific environment and population allows for a detailed exploration of children’s unique challenges in that setting. Furthermore, by highlighting the importance of communication, the study underscores the need for more inclusive healthcare practices. Though not widely generalisable, the findings provide a valuable foundation for future research, particularly in exploring the needs and experiences of children in similar healthcare environments.

### 4.2. Implications Practice

The study’s findings emphasize the importance of pediatric healthcare providers actively involving hospitalized children in their care. Children express a strong desire to participate, making it crucial for nurses and providers to keep them updated on their care plans, explain procedures, and involve them in decisions whenever possible. This approach respects the child’s autonomy and reduces anxiety caused by uncertainty.

The practice of child-centered care, which focuses on the child’s perspective in collaboration with their family, is gaining recognition alongside traditional family-centered care. Rather than competing, these approaches complement each other by prioritising the child’s voice while still considering the family’s role. The Pediatric Bill of Rights [15] reinforces this by outlining children’s rights to information, support, and care choices.

Hospitalized children also desire to maintain their childlike activities, such as play and social interaction, even during illness. Pediatric nurses play a vital role in supporting this need by engaging with children beyond medical duties, thus fostering a more comforting and less restrictive environment. Moreover, understanding the child’s world and avoiding an adult-centric perspective helps providers connect with children on their own level.

The active involvement of children in their care not only benefits the children but also enhances nursing practice. By incorporating the child’s input, nurses can develop more effective and personalized care plans. This collaborative approach encourages professional growth and satisfaction among nurses, as they witness the positive impact of their efforts on the child’s well-being. Furthermore, it promotes a culture of empathy and respect within the healthcare team, ultimately leading to improved patient outcomes

## 5. Conclusions

Integrating children’s verbal reports provides valuable insights into their hospitalization experiences. Addressing issues related to emotional impact, the need for parental presence, communication needs, and the role of patient companions through innovative approaches can enhance pediatric care. Recommended strategies include improving communication, supporting family involvement, and utilizing creative interventions to address emotional needs. Future research should explore the long-term impacts of child-centered care on children’s psychological and emotional well-being post-hospitalization. Additionally, studies could investigate the effectiveness of various communication strategies used by healthcare providers to involve children in their care. Research on the integration of play and social activities in hospital settings and their effects on recovery and overall patient satisfaction would also be valuable. Evaluating and implementing these strategies to enhance children’s hospitalization experiences and explore how innovations can be integrated into clinical practice is crucial.

## Figures and Tables

**Table 1:** Specific interview questions.

“How did it feel being in a place that wasn’t your home?”
“What did you think when you saw all the tubes and machines? Did they scare you?”
“Did you know what all those things were for?”
“Did you ever see or hear weird things? How did that make you feel? Did you feel lonely in the hospital? Why?”
“How did you feel when you missed them?”
“Was it difficult to sleep in the hospital?
“What bothered you?”
“What things made you feel nervous?”
“Did the tubes or machines hurt? What was the worst?”
“Did it feel you had to stay in one position for too long? Was it difficult to tell the doctors or nurses how you felt?”
“How did you tell them what you needed if you couldn’t talk?”
“Did you understand what the doctors or nurses were doing?”
“They should explain things better?”
“Was there a nurse or doctor that made you feel better?

**Table 2:** Participants Profiles.

Participants	Gender	Age	Social Background Lives	Illness	Days Hospital
1	F	9	Parents and sister	Asthma	6
2	F	9	Mother	Abdominalpain	7
3	F	10	Parents and brother	Pneumonia	10
4	M	11	Parents/brothers	Bronchitis	5
5	M	11	Parents	Migraine	6
6	M	12	Mother and sister	Asthma	6
7	F	12	Parents	Peritonitis	10
8	M	13	Parents/2 sisters	Traumatic brain	15
9	F	10	Parents/sister	Pneumonia	8
10	M	10	Parents	Appendicitis	7

All data is based on patient records and interview logs.

**Table 3:** Themes and subthemes.

Themes	Subthemes
3.1. Emotions and Feelings	Fear of Death and Unfamiliar Hospital Setting. Anxiety about Medical Equipment
	Loneliness and Isolation
	Sensory Overload
3.2. Experiences of Pain and Discomfort	Disturbances from Hospital Environment. Medical Procedures and Improper Positioning
3.3. Interaction and Communication with Medical Staff	Communication Inability
	Lack of Information
	Positive Interaction with Nurses

## Data Availability

The data presented in this study are available upon reasonable request and subject to privacy. For further information on how to submit a request for data access, please get in touch with the corresponding author.

## Supplementary Materials

The following supporting information can be downloaded at: https://www.mdpi.com/article/10.3390/nursrep14040228/s1, Table S1: Standards for Reporting Qualitative Research (SRQR).

## Public Involvement Statement

There is no public involvement in any aspect of this research.

## Guidelines and Standards Statement

This manuscript was drafted against the consolidated criteria for reporting qualitative research (COREQ) for qualitative research.
